# A Survey of Laboratory and Statistical Issues Related to Farmworker Exposure Studies

**DOI:** 10.1289/ehp.8528

**Published:** 2006-02-16

**Authors:** Dana B. Barr, Doug Landsittel, Marcia Nishioka, Kent Thomas, Brian Curwin, James Raymer, Kirby C. Donnelly, Linda McCauley, P. Barry Ryan

**Affiliations:** 1 National Center for Environmental Health, Centers for Disease Control and Prevention, Atlanta, Georgia, USA; 2 Department of Mathematics and Computer Science, Duquesne University, Pittsburgh, Pennsylvania, USA; 3 Battelle Memorial Institute, Columbus, Ohio, USA; 4 National Exposure Research Laboratory, Office of Research and Development, U.S. Environmental Protection Agency, Research Triangle Park, North Carolina, USA; 5 National Institute for Occupational Safety and Health, Centers for Disease Control and Prevention, Cincinnati, Ohio, USA; 6 RTI International, Research Triangle Park, North Carolina, USA; 7 Texas A&M University System Health Science Center, College Station, Texas, USA; 8 School of Human Environmental Sciences, University of Pennsylvania, Philadelphia, Pennsylvania, USA; 9 Rollins School of Public Health, Emory University, Atlanta, Georgia, USA

**Keywords:** analytical methodology, biomarkers, laboratory, limit of detection, omics, quality control, sample size, statistics

## Abstract

Developing internally valid, and perhaps generalizable, farmworker exposure studies is a complex process that involves many statistical and laboratory considerations. Statistics are an integral component of each study beginning with the design stage and continuing to the final data analysis and interpretation. Similarly, data quality plays a significant role in the overall value of the study. Data quality can be derived from several experimental parameters including statistical design of the study and quality of environmental and biological analytical measurements. We discuss statistical and analytic issues that should be addressed in every farmworker study. These issues include study design and sample size determination, analytical methods and quality control and assurance, treatment of missing data or data below the method’s limits of detection, and post-hoc analyses of data from multiple studies.

Developing internally valid, and perhaps generalizable, farmworker exposure studies is a complex process that involves many statistical and laboratory considerations. Statistics are an integral component of each study beginning with the design stage and continuing to the final data analysis and interpretation. Similarly, data quality plays a significant role in the overall value of the study. Data quality can be derived from several experimental parameters, including statistical design of the study and quality of environmental and biological analytical measurements. Because these issues are so intricately intertwined and affect almost all aspects of the study, we chose to discuss these issues together. This survey of issues is intended as a guide or resource for epidemiologists, exposure assessors, and others who plan to undertake a study involving a highly mobile farmworker population.

A large number of different statistical issues arise in the development and analysis of farmworker pesticide exposures and resulting data. These discussions can be generally categorized as relating to *a*) sampling and design (i.e., pre-data collection) issues; *b*) laboratory quality issues; *c*) missing or misclassified data and subsequent biases, including limits of detection (LODs; i.e., primarily preanalysis issues); *d*) data reporting and reliability issues (i.e., primarily descriptive statistical issues); *e*) extensions to standard statistical models and analyses (i.e., primarily inferential statistical issues); and *f*) post hoc analyses and combining study results. It is also important to use the proper interpretation and context for analysis, including consideration of scientific information and understanding the direction of test statistics.

## Statistical Issues at the Design Stage

A primary concern of studies associated with farmworker exposures is choosing the appropriate sampling strategy. Ideally, the objective of any sampling strategy is to collect data that are representative of the study population (Rothman and Greenland 1998). However, representativeness is often difficult to obtain. Although statistical representativeness is certainly a desirable attribute, it is neither a necessary nor a sufficient condition for a well-designed investigation. For example, case–control studies are rarely statistically representative. For cases in which very little information is available, it may be impossible to know whether the study is representative of the population. In such conditions, an exploratory investigation is warranted. Convenience or anecdotal samples may give very useful information. Because responses from convenience samples are likely to be better than that from a representative sample, they may actually be more “representative.” Data gathered from such an investigation can prove invaluable in developing a better design in a representative sample.

Representativeness is especially challenging in the setting of farmworkers because of the numerous barriers that exist in defining the populations, temporal and geographic trends in exposures, range of job activities for a given worker, worker mobility, and other factors related to use/exposure to toxic chemicals, viruses, sun, co-pollutants, and noise. Perfectly random sampling across all relevant factors is therefore almost universally impractical; some form of convenience sampling is typically adopted in practice. However, the implication of such a practice is that the resulting sample is not representative of the intended population, and the consequences of such actions are not obvious without a specific understanding of how the sample’s characteristics differ from those of the population. One common sampling approach in this setting is to sample (either entirely or at least oversample) known or anticipated “hot spots” of exposure. Unless samples are weighted for the oversampling, this approach may potentially bias the resulting exposure estimates because it systematically overestimates the exposures.

Determining the specific sampling strategy depends on the sources of variability (Kish 1965). For example, collecting 10 samples from 10 people on the same farm is not equivalent to 10 samples from 10 different people on 10 different farms; the latter case represents 10 completely independent samples. Sources of variability include a wide range of factors in this setting, including within-family and genetic sources of correlation, correlations within geographic regions, similarities among workers at a given farm, and other sources of correlation and clustering (Fleming et al. 1992; McKnight et al. 1996). Standard statistical procedures assume independent observations. Treating correlated data as independent will lead to biased *p*-values by under- or overestimating the variability of resulting test statistics (Smith et al. 1992). In the following sections, we discuss the resulting statistical issues and possible approaches in more detail as they relate to prospective cohort studies.

### Sample-size determination

In addition to determining the sampling strategy, it is important to collect data on a sufficient number of subjects and to collect a sufficient number of repeated measures on a given subject (Gilbert 1987; Kish 1965). The determination of an adequate sample size and an adequate number of repeated measures is a complex process in this setting because of the factors that contribute to clustering and correlation. In addition, one must consider the temporal and job-associated factors that lead to within-subject variability across repeated measures on an individual. For example, in cases where farmworker tasks vary substantially across different times of day or seasons, repeated samples are necessary to capture the resulting variability. The greater the variability across time, or across tasks, the greater the need for repeated measures on a given subject.

Therefore, one must determine a sampling strategy and appropriate sample size requirements, including within-subject repeat sampling, that maximize the resulting amount of information achieved with respect to the within-subject versus between-subject variability for the given problem (Ryan et al. 2000). Consideration of even a few of these factors commonly leads to complex analytical, theoretical, or simulation-based approaches for determining the appropriate sample sizes (Hendricks et al. 1996; Rochon 1998). However, both collection and measurement costs often limit the ability to collect a large series of longitudinal samples on single or multiple individuals (Rennolls 1991). Some farmworker populations are also highly mobile and perform many different tasks on a single farm or across different kinds of farms, creating additional difficulties in longitudinal exposure studies (Quandt et al. 2002).

## Analytical Measurements in Pesticide Exposure Studies

Once a sampling strategy has been identified and appropriately implemented, individual or composite samples are typically analyzed for pesticides or their metabolites. Analyses of environmental and biological samples involve the same general steps, although some matrix-dependent refinement is often necessary.

The diversity of exposure scenarios monitored by researchers, coupled with the diversity of pesticides in use, presents an extremely complex picture for harmonization of sampling and data analysis approaches. In addition, a wide range of analytical techniques can be considered. For example, some researchers examining the exposure of farm communities to one or two widely used pesticides may need only a single- or dual-residue method (Fenske et al. 2002), whereas others who are looking at exposures to a wider range of pesticides, and assessing exposures to as many as 20, 40, or even 100 different pesticides, require multiresidue methods (Rudel et al. 2001). Some exposure scenarios involving high-level, episodic exposures may require analytical methods with only high levels of detection (e.g., micrograms per milliliter in the sample extract) (Lewis et al. 2001). Other exposure scenarios involving low-level chronic exposures often require analytical methods with trace detection levels (i.e., nanograms per milliliter or lower in sample extracts) detection limits (Hartge et al. 2005). Because sample collection methods will influence the laboratory analysis, all exposure assessment studies should start with contacting the appropriate laboratory to ensure compatible sample collection methods.

Most methods for measuring pesticides or their metabolites in either environmental or biological samples use a sample preparation step to isolate the target chemical(s) from the matrix, a cleanup step to remove unwanted coextractants, an instrumental analysis technique with a selective detection system for detection and quantification, data processing, and quality assurance (QA) processes (Barr and Needham 2002). Some of the options for these main steps are discussed below.

### Sample extraction

The initial extraction from a solid matrix (e.g., dust, wipes, soil) is usually achieved using a solvent extraction technique. Choices include Soxhlet extraction, blender homogenization, sonication, and accelerated solvent extraction (ASE) (Curwin et al. 2003; Rudel et al. 2003; Wilson et al. 2003). Soxhlet extraction and blender homogenization use partitioning of the analyte into the solvent as the means for extraction. With sonication, ultrasonic energy is used to help drive the analytes into solution; with the ASE technique, both elevated temperature and pressure are used to assist in the solubilization of the analyte(s) in the solvent. Advantages of ASE include lower solvent use, faster extraction times, and less apparatus to clean. Dust, soil, dermal and surface wipes, food, air sorbents, and air filters have been extracted successfully with this technique. With any of these techniques, the choice of solvent is the critical factor. Solvents are generally matched in polarity to the target analyte(s) to maximize extraction of the target analyte(s) and minimize extraction of potential matrix interferences.

The extraction of analytes from an aqueous matrix (e.g., water, beverages, urine) is usually accomplished using either solvent partitioning into an immiscible solvent, such as dichloromethane, or solid-phase extraction (SPE). With an SPE method, analytes partition from the liquid phase into or onto the surface of solid sorbent particles. One of the most frequently used SPE phases is C_18_; this sorbent is composed of octadecyl carbon chains chemically bound to a silica surface. Several relatively new SPE sorbents (e.g., OASIS, NEXUS, STRATA) offer a mixed polarity polymerized phase to allow for simultaneous extraction of diverse chemicals.

### Sample cleanup

Cleanup of the sample extract can be achieved using any one of various chromatographic techniques. Many analytical methods for trace analysis require this step for proper operation. Although there may not be direct interferences from the matrix to a given analyte in the final detection method, a significant amount of co-extracted material may have a deleterious effect on instrument performance. This degradation in instrument performance is seen as a loss of sensitivity, loss of chromatographic resolution, or instability of calibration. Semipreparative cleanup steps such as precipitation, liquid–liquid partitioning, and gel permeation chromatography (GPC) can be used to remove large quantities of co-extracted material. For instance, GPC is routinely used to remove lipids from dietary/food and blood samples; however, this technique has also been used to remove these same types of compounds from house dust (Moate et al. 2002). The SPE technique described above for extraction can also be used as a chromatographic cleanup step (Nishioka et al. 2001). In addition to using a smaller amount of solvent, these cartridges can be stacked to optimize tandem cleanup steps, thus cutting cleanup times considerably.

The sample preparation and cleanup steps are usually the most common source of analytical error, whether systematic or random, because the sample is frequently handled by humans. Automated sample preparation techniques, such as automated SPE or GPC, are usually more precise. If the chemical is inherently incompatible with the analytical system that follows, a chemical derivatization (Bravo et al. 2002, 2004; Hardt and Angerer 2000; Hill et al. 1995; Lin et al. 2002; Moate et al. 1999; Stalikas and Pilidis 2000) or reduction procedure may also be required. The addition of steps into the sample preparation procedure usually increases the overall imprecision of the method.

### Analytic detection

The third step of the method involves detection and quantification. Although there is usually a semipreparative chromatographic cleanup step in the method, very few methods allow detection of an analyte at trace levels without high-resolution chromatography preceding the detector. Environmental and biological samples are simply too complex. Typical detection methods couple a gas chromatograph (GC) with a mass spectrometer (MS) or with a selective detector (e.g., electron capture detector, nitrogen phosphorus detector), or couple a high-performance liquid chromatograph with MS or tandem MS (MS-MS). The MS-based techniques rely on several features of the system to confirm detection: the retention time (from the chromatographic part of the system) and detection of two or three diagnostic ions that are in the correct ratio to each other. The diagnostic ions must co-maximize at the correct retention time in the proper ratio for an analyte to be considered detected. In the tandem MS-MS techniques where a selected diagnostic ion is passed from one MS through a collision chamber to the next MS, an additional confirmation of identification is obtained by having the transition from a characteristic “precursor ion” to a “product ion” at the correct retention time. This specificity of an MS-MS analysis can be very useful for analytes that have relatively low-mass ions with frequent interferences. Although MS-based systems are very reliable and especially useful for multiresidue methods, a specific detector such as a nitrogen phosphorous detector for organophosphate insecticides or an electron capture detector for organochlorine pesticides can often be used for single analyte detection or for multiresidue methods that cover a narrow compound class range.

Another analytic technique that is often employed for measuring pesticides is immunoassays (IAs) (Biagini et al. 1995; Brady et al. 1989; Dzgoev et al. 1999; Lyubimov et al. 2000; Sanderson et al. 1995; Thurman and Aga 2001). For this technique, a sample preparation step to isolate the chemical from the matrix may or may not be used. Many IAs are commercially available for selected chemicals for some types of sample matrices. However, the development of an IA for a new chemical is a lengthy process that typically requires the generation and isolation of antibodies and then the development of the assay itself. Usually ultraviolet, fluorescence, or radioactivity detection is used for the assays. IAs may be very specific for a given chemical, or they may have a great deal of cross-reactivity that can limit their utility for single pesticide identification. This cross-reactivity may allow assessment of exposure to a class of chemically related pesticides (Kaufman and Clower 1995). The LODs for IAs can vary widely; however, many have adequate sensitivity for measuring low-level exposures, but most are targeted at measuring occupational exposures. The imprecision usually ranges from 10 to 15%, and the throughput is usually quite high (> 100 samples per day).

The quantification of analytes can be accomplished using external calibration, the internal standard (IS) method, or, if MS is used for detection, the isotope dilution method. With the IS method, the IS is added at a fixed level to samples and standards. The relative ratio of response for analyte to IS is used to even out minor variations in injected volume, volatilization in the injector, transport from injector to the column, and column activity in GC-MS analyses. Where feasible, the isotope dilution method is preferable because the addition of the stable isotope at the beginning of the method (at extraction) can be used to account for all analytical method losses and ionization effects. Unfortunately, relatively few compounds that contain stable isotopes are available, and most are costly. In this case, the addition of surrogate recovery standards (SRSs) at the point of extraction and measurement of the recovery of these SRSs is used to assess (and possibly correct for) the method performance on a sample-by-sample basis. Information on extraction efficiency can be used to adjust the reported values to compare values across studies.

### Method performance

Although the pesticides and analytical methods may vary substantially, documented method performance is fundamental to all studies. Documenting method performance entails assessing at a minimum the accuracy, precision, and LOD. Additional useful factors to assess performance include storage stability, ruggedness, and operational range. The accuracy is assessed by measuring the percent relative recovery of analytes. To assess relative recovery, a known concentration of each analyte is added to unused samples or sample matrix to create spiked samples. The amount of analytes added should be representative of the amounts likely to be found in actual field samples. For matrices such as soil, dust, or urine, the actual matrix can be readily obtained. Care must be taken to ensure that background levels of the analytes are sufficiently low so that they do not interfere with low-level spikes to the matrix (typically, spike levels need to be about 4–5 times greater than a background level). For matrices such as dermal wipes, there is a critical need to prepare a matrix that is as similar as possible to the field matrix. To accomplish this for a manual field harvester exposure study, Boeniger et al. (Boeniger MF, Nishioka MG, Carreon T, Sanderson W, unpublished data) ground up several of the typical commodities (e.g., cauliflower leaves, strawberries, lettuce), mixed this pulp with soil, and applied the mixture to pieces of pig skin obtained from the local rendering plant. This “dirty” pigskin was wiped with a wipe moistened with isopropyl alcohol, and the wipe was then inoculated with the pesticides of interest before extraction to provide a robust assessment of method performance (Boeniger MF, Nishioka MG, Carreon T, Sanderson W, unpublished data). Pristine gauze wipes would not have been an accurate reflection of the challenges of the field samples. In many instances, however, it is difficult to simulate all of the materials in a complex matrix unless the matrix both is well characterized and provides repeatable measurements, which is not always the case with farming environments and farmworkers. The precision of the method is then assessed via the relative standard deviation of replicate spike recovery values in the chosen field matrix. Analyte recoveries are usually lower at lower analyte spike levels, particularly approaching the LOD, and this aspect of the method should be assessed in case global recovery adjustments are needed. The variability of relative recoveries among discrete matrix samples should be evaluated.

Various terms and definitions are used to describe the lowest level of accurate identification or quantification: method quantification limit, method detection limit (MDL), limit of quantification, and LOD. Because different definitions exist for each, it is essential to state the method or definition that is used. Given that samples cannot be quantified below a certain level, any nondetected analyte should be reported as < LOD or < MDL, with those limits stated. The frequency of detection is always a function of the method LOD. In later sections we discuss in more detail the issue of calculating or imputing the LOD.

### Quality assurance/quality control

Important elements of the analytical effort also include planning the quality control (QC) samples that are generated in the field and the laboratory. These samples are distinct from, and do not replace, QA samples that are typically analyzed by two different laboratories to help uncover potential bias in the data. It is preferable to use field QC samples, in addition to laboratory QC samples, to qualify method performance, because those samples will have undergone all the handling and storage of the actual field samples. In the field, QC samples include field blanks to assess whether samples have been contaminated in the field; field spikes to assess whether the analyte concentration changes during collection, shipping, and handling; and field duplicates (or replicates) to assess the variability of the concentration in the matrix. Field blanks, field spikes, and duplicates should make up 5–10% of the total field samples, with a minimum of at least three to five of each type. Laboratory QC samples should be included to ensure the laboratory portion of the study is operating correctly. Solvent method blanks, or matrix blanks, need to be processed with each sample set generated in the laboratory to ensure no laboratory contamination. Fortified method blanks and laboratory matrix blanks allow assessment of potential analyte losses from laboratory procedures.

Further QA/QC can also be incorporated into the methodology, especially for well-established methods used on a routine basis (Barr and Needham 2002). QA/QC programs typically comprise formal detailed protocols to ensure adherence to a given method and multiple testing procedures that easily allow the detection of systematic failures in the methodology (Barr and Needham 2002). The testing procedures can include proficiency testing to ensure accuracy as measured against a known reference material, repeat measurements of known matrix materials (laboratory QC) to confirm the validity of an analytical run and to measure analytical precision, “round robin” studies to confirm reproducible measurements among laboratories analyzing for pesticides or metabolites, regular verification of instrument calibration, daily assurance of minimal laboratory contamination by analyzing “blank” samples, and cross-validations to ensure that multiple analysts and instruments obtain similar analytical values. Many laboratories have adopted comprehensive QA/QC programs to ensure valid measurement results (Needham et al. 1983; Schaller et al. 1995). For instance, some public health laboratories in the United States have been certified by the Center for Medicare and Medicaid Services to comply with all QA/QC parameters outlined in the Clinical Laboratory Improvement Amendment of 1988 (1988), and many other laboratories have received International Standard Organization quality registrations. Studies conducted by pesticide industries for U.S. Environmental Protection Agency compliance are performed under Good Laboratory Practice protocols. The Federal Republic of Germany has chosen to implement a rigorous internal and external QA program for biological analyses (Lehnert et al. 1999; Schaller et al. 1991, 1995). Many parameters for implementing or improving a QA program have been published (Schaller et al. 1991; Taylor 1987; Westgard 2002).

Because pesticides are often measured using expensive instrumentation and require highly trained analysts, these measurements are usually costly. The most selective and sensitive methods are usually the most complex and can range in cost from $100 to $500 per sample analyzed. Many of the analyses are multi-analyte panels, so the cost per analyte per sample is much more reasonable. IAs are less specific and less complex; therefore, their cost is usually less than $50 per test. However, usually only one chemical can be measured per test, and new chemicals cannot be easily incorporated into the method. IAs may have potential also for screening samples to determine the necessity of further testing.

## Statistical Issues at the Analysis Stage

Many statistical issues arise in the analysis of farmworker and farmworker family pesticide exposures and related data. In the following paragraphs, we briefly survey these issues.

### Missing data and LOD issues

The general issue of missing data often represents a substantial consideration for analysis of pesticide exposures. They can pose serious problems in the data study by reducing statistical power and the statistical efficiency of estimation and may ultimately result in bias of estimates or distortion of the nominal type I error in tests. Potential approaches for handling missing data include use of complete data only, standard imputation, multiple imputation, and/or propensity score adjustment (Baker et al. 2006). The most simplistic approach of using only subjects with complete data reduces the effective sample size and subsequently reduces statistical power. To address this concern, a common approach is to then impute the mean value for any missing data. Doing so, however, underestimates the variability of the data (because a single value is imputed for multiple points) and subsequently produces liberal estimates of statistical significance. To circumvent this limitation, multiple imputation (Little and Rubin 2002) uses sampling from a distribution of values to appropriately estimate the variability and statistical significance. For any of these approaches, however, one must consider the underlying mechanism (Rubin 1976) leading to missing data. For instance, the most basic assumption of missing at random is typically unrealistic, especially in the context of assessing pesticide exposures; more complex mechanisms may invalidate results, producing biased estimates and inferences. One approach to evaluate potential bias is to model the probability of being missing and incorporate the results as a model covariate (Baker et al. 2006). In summary, a review of these issues and potential approaches to addressing missing data is necessary before beginning any subsequent analyses.

A special classification of missing data is those that fall below the analytical method LOD (i.e., censored data). In many studies, pesticide concentrations are often low, and frequently in a set of samples, some will have no detectable levels of a pesticide. The choice of what to do with this information is not straightforward, and investigators may choose to assume that a nondetected level is 0 or some value between 0 and the analytic LOD. The study design choices related to this issue, such as whether to collect a convenience sample or to oversample “high-end” individuals (and then adjust the data using statistical weights), are important considerations that cannot always be predicted if there are few data on a specific active ingredient or few representative measurements in the environmental medium of interest. The use of any single specific value as a substitute for missing values has potentially significant implications on measures of central tendency or variance, as do more rigorous statistical treatments (e.g., bootstrap methods or Bayesian approaches to approximate the censored distributions) that may provide more scientifically defensible answers but may still not provide insight into exposure–effect associations or differences between groups of interest. A recent report examining the effect of nondetectable values on exposure–disease associations in an epidemiologic study of non-Hodgkin lymphoma is a good illustration of this point (Lubin et al. 2004). The general approach has been to use one-half of the LOD; however, data have also been analyzed excluding the samples below the LOD, or ranges have been given. Hornung and Reed (1990) suggest that when most of the data are below the LOD, reporting a mean and standard deviation is a questionable practice and that a better description of the data would be to simply report the percentage of samples below the LOD and the range of the remaining samples.

Measurements below the LOD pose a significant challenge in biomonitoring and are not easily reconciled. The problem can be generally classified as either measuring a signal that falls within the range of instrument error or failure to measure a potentially informative signal at some low range of the data. As public health concerns continue to emphasize protection against even small exposures, detection limits represent an increasingly significant concept. The definition of a LOD, however, has not been well defined and varies conceptually and practically between studies (Currie 1988). In general, the LOD may be based on repeated background measurements (and subsequent prediction intervals), knowledge about the technical limitations of the monitoring device, or other knowledge about the nature of the measurements. Such data can be analyzed via several different strategies. Possible approaches include treating all undetectable measurements as zero, assuming all undetectable measurements are less than the minimum detectable data or assuming all undetectable measurements are less than some specified threshold.

Using a simple imputed value for each undetectable measurement, such as the one-half the LOD, can lead to bias and loss of power (Hughes 2000). He discusses the resulting bias and power and incorporation of standard survival methods for more appropriately treating the undetectable measurements as censored values. Assumptions concerning existence of a specific threshold can also be flawed because an actual separation may not exist between detectable and undetectable measurements. To address this issue, a distribution can be assigned to the probability of detection for a given measurement (Cohen and Ryan 1989). Lambert et al. (1991) used local logistic regression to estimate these probabilities and determine whether a reliable threshold existed and, if it did, estimated that threshold.

Pesticides are ubiquitous in our environment, and those that persist are present in low concentrations in many areas not subject to pesticide treatment. With the advent of new, high-sensitivity methods, the LOD problem is often superseded by a determination of whether a measured value exceeds what might be considered a “background” concentration. Variation in background levels, as well as background concentrations themselves, can be used to identify a threshold for “elevated” levels that minimizes both type I (false-positive) and type II (false-negative) errors. A variety of statistical procedures can be applied to analyze subsequent data, including both parametric and nonparametric methods. Shumway et al. (2000), for example, review the use of maximum likelihood estimation and regression on order statistics for potentially nonnormal censored data and propose corresponding exact statistics. Others define simple nonparametric statistics based on an average of background measurements (Linnet and Kondratovich 2004).

Censoring may also be a problem for such data. Samples may be below the LOD either because there is no pesticide to be measured or because the method used to assess the pesticide concentration is not sufficiently sensitive. Assessing normality in such situations is often complicated but can be evaluated via methods such as correlating the Kaplan-Meier estimates with normal probability plots (Hawkins and Gehlert 2000). Hornung and Reed (1990) proposed three methods for assessing summary statistics in the presence of significant left-censoring. The best method invokes a complex maximum likelihood statistical imputation method. However, in many cases this is not necessary. If the degree of left-censoring is not large and the data are highly skewed (geometric standard deviation ≥ 3), then substitution of the LOD/2 for the censored data is suggested. For less skewed data, substitution of the LOD divided by the square root of 2 produces reasonable estimates of summary statistics. Each of these methods assumes a certain character for the distribution. If the data are skewed, as most pesticide data are likely to be, the error estimates for the mean and any estimate of the variance are not likely to be influenced strongly by the selection of LOD method; the uncertainty in the parameter estimates is likely to be larger than the effect of the LOD choice. However, in the limit of an infinite number of samples, the results will still be biased. Deciding whether to use parametric or nonparametric methods, however, depends in part on the assessment of normality, which can be complicated by left-censoring. In this setting, normality can be evaluated by correlating the Kaplan-Meier estimates with normal probability plots (Hawkins and Gehlert 2000).

The problem of undetectable measurements may be further complicated by the existence of multiple detection limits across samples, which may, for instance, result from collection at different time points, measurement by different instruments or procedures, or confounding by systematic instrument variation. Insufficient research has been published on this topic because most methods assume a single threshold, although some of the previously described methodology generalizes to multiple censoring points. Hawkins and Gehlert (2000), for example, investigated the cases of single and double censoring for their method. Further methods for handling values below the LODs should be evaluated and applied consistently across studies.

### Potential misclassification bias

A separate but related set of issues to missing data is the problem of measurement error, subsequent misclassification, and resulting bias. Measurement error of some type often arises in observational studies because of the use of surrogate variables (e.g., overall job task), self-reported behaviors (e.g., frequency of pesticide use), or other inexact measurements (Kauppinen 1994). Even when the surrogate measurements are relatively sensitive to the exposure of interest, substantial bias can result when making inferences about some intervention of other risk factors (Gardner et al. 2000). Various regression models have been proposed as adjustment methods for these and other problems related to measurement error (Lyles and Kupper 1997).

### Data reporting issues

Biomonitoring and environmental data have been reported in the literature in a variety of ways and are a function of the subject population, sample matrix, analytical methods, and data analysis. Currently, no standard way of reporting these data exists, making it difficult to compare data among studies. Four general issues of concern have emerged: normalization, descriptive statistics, demographic categorization, and data censorship. Discussion of inter- and intralaboratory reliability is also included in this section.

#### Normalization of concentration units

Concentrations of pesticides or their metabolites in biological samples are usually reported as the weight of analyte per volume sampled (e.g., milligrams per liter). When spot urine samples are collected, the hydration state of the study subject should be considered. Urine volumes vary widely and influence the concentration of pesticide in the urine, making comparisons difficult if pesticide concentration is reported in mass per volume units. Although urine volume can vary as much as 4-fold, the mass of solid materials dissolved in urine has only a 2-fold variability (Talaska 2003). Normalizing the urine results by adjusting the reported pesticide concentration by the amount of specific dissolved materials may provide a more reliable concentration. The most common way this is done is by adjusting the pesticide concentration in urine by the amount of creatinine in the urine sample; however age, sex, and racial/ethnic differences in creatinine excretion complicate comparison of creatinine-adjusted values in diverse populations (Barr et al. 2005). Creatinine adjustment is accomplished by dividing the pesticide concentration (in amounts per liter units) by creatinine concentration (in grams per liter units) to yield a concentration in amount of pesticide per gram of creatinine units. Creatinine-adjusted pesticide concentrations have also been reported as milligrams of pesticide per micromole of creatinine or micromoles of pesticide per gram of creatinine, further complicating the comparability of the data across studies. To conform with most data published in the literature, micrograms of pesticide per gram of creatinine units are recommended.

Another method for adjusting pesticide concentration in urine is to adjust for the specific gravity of the urine, typically normalized to a specific gravity of 1.024. The adjusted values are reported in micrograms per liter units, similar to unadjusted data, although the dilution or concentration of urine is taken into account. Because specific gravity measurements appear in the numerator and the denominator during adjustment, their units effectively cancel out, leaving the original units of measure.

Often, the reported data are presented as both unadjusted and adjusted values. Presenting both the unadjusted and adjusted values helps the reader to compare the data with other studies and determine the reliability of the adjustment; thus, presentation of both values is recommended.

#### Descriptive statistics

Pesticide biomonitoring and environmental data have been reported as ranges, geometric means, arithmetic means, medians, and distribution percentiles. Generally, these data are skewed to the right and are often log-normally distributed. For this reason, the geometric mean is most often reported. Pesticide data are often log-transformed to perform statistical analyses and modeling. If the arithmetic mean and standard deviation are given, then there is an implicit assumption of normality because these statistics are not helpful in the case of nonnormally distributed data. The median is a measure of central tendency regardless of the analytical form of the distribution. Knowing the type of distribution, then, is not as important when reporting median data. If a large proportion of the sample measurements are below the LOD of the analysis method, then the range of the data is often reported without the reporting of any type of central tendency value. Less commonly, pesticide data may be reported in distribution percentiles such as the 50th, 90th, 95th, or 99th percentile. This general nonparametric approach is desirable, especially in studies with pesticides where a significant fraction of the data may be below the LOD.

#### Demographic categorization

People of different ages metabolize pesticides differently, and different racial and ethnic groups may have genetic susceptibilities, making the interpretation of pesticide biomonitoring data difficult, particularly when trying to associate exposure with health effects. Data have been reported for both adults and children separately. However, there are substantial differences in child physiology and behavior at different ages that can have a substantial impact on exposure to pesticides. Toddlers and young children may have increased contact with contaminated surfaces and soil and increased hand or object-to-mouth behaviors, whereas older children may perform work or chores that can result in higher exposures. Among adults, males and females can have different exposures and response to the exposures. Older adults may also have different exposures and responses to these exposures than younger adults. Therefore, for pesticide data comparability, it would be appropriate to define age categories, sex categories, and racial/ethnic categories and report data specific to these categories as well as for the overall population group.

#### Assessing inter- and intralaboratory reliability

A further issue in summarizing data is assessing reliability across both different analytical methods and different laboratories. Standard methods such as κ-statistics (Bloch and Kraemer 1989) or intraclass correlations (Rosner 2005) can be employed to quantify reliability. The κ-statistic, which is appropriate for categorical data, measures the degree of agreement beyond chance. The intraclass correlation measures the degree of reproducibility between repeated observations within the same subject. These statistics should be used in favor of, or in addition to, reporting simple correlations or other statistics that do not take within-subject variability into account.

### Extensions to standard statistical models and analyses

One of the previously mentioned complications of evaluating farmworker exposures is the potential for correlation between observations, such as repeated measurements on a given worker or clustering within subgroups of the populations. Ignoring such correlation, that is, treating each measurement as an independent observation, underestimates the variability of the data, thus leading to overestimated precision and significance levels. A wide range of methodologies, however, exist for appropriately analyzing correlated and longitudinal data (Diggle et al. 2002). The most common approach is to adjust variance estimates via generalized estimating equations (Liang and Zeger 1986), thus allowing for appropriate estimation and inference, even when the exact structure of dependence is not known. Another approach is to treat the individual subject as a random effect and explicitly model the correlation structure through mixed effects regression models (Laird and Ware 1982). A number of publications have addressed possible methodologies for quantifying sources of variability specific to given occupational settings (Kromhout et al. 1993; Peretz 2002; Preisser et al. 2003; Rappaport 1995), many of which deal directly or indirectly with exposure assessment and/or other applications relevant to studying pesticides and associated exposures or morbidity.

#### Data reduction and multiple testing

Another specific problem that arises with increasing frequency among farmworker pesticide exposure studies is determination of appropriate statistical methods for analyzing high-dimensional data and a large volume of associated statistical tests. The need for data reduction occurs when the number of measurements taken cannot be supported by the observed sample size or cannot be handled in a logical scientific framework. One can generally take the approach of either data reduction (i.e., formulating a smaller number of variables) or multiple testing (i.e. adjusting for the case of many significance tests and possibly false significant results).

For data reduction, one typically calculates some function(s) of the original variables that captures a high degree of their variability in a smaller number of variables. The most common approach for data reduction is principal component analysis, that is, calculating independent linear combinations of the original variables that maximize the resulting variability (Myers 1990). One typically retains the smallest number of principal components needed to capture at least 80–90% of the variability (Cohen et al. 1989).

Regarding the alternative approach of multiple testing, a wide variety of methods is now available to appropriately adjust the significance level in the presence of a large number of statistical tests. The most crude, and overly conservative, approach is the Bonferroni correction, which divides the critical significance level (usually 0.05) by the number of tests being conducted. This greatly reduces the probability of a false significant result but also leads to a much reduced statistical power. Other alternative measures have been more recently developed, such as the Benjamini-Hochberg method, which iteratively adjusts the significance levels based on minimizing the expected proportion of false significant results (Benjamini et al. 2001). The Benjamini-Hochberg method achieves nearly the same control of false significant results while greatly improving statistical power compared with the Bonferroni method. A wide range of additional methods can be generally categorized as single-step, step-down, or step-up methods; such methods have gained significant popularity in the context of microarray data analysis (Dudoit et al. 2003) and may be of similar interest for future researchers performing farmworker pesticide studies using high-throughput methods. In cases where there are multiple outcomes and/or specific alternative hypotheses about the nature of exposures and their effects, global tests such as O’Brien’s test (1984) may be more appropriate and statistically powerful.

Despite their intuitive appeal for reducing the dimensionality of large-scale exposure data, the use of multiple testing methods has been frequently criticized in epidemiologic settings. Savitz and Olshan (1995), for example, discuss the use of multiple testing in terms of appropriately interpreting epidemiologic studies. Rather than simply limiting findings based on a strict *p*-value criterion, they recommend a careful evaluation of study results that focuses specifically on a well-defined question. Rothman (1990) argues more fervently against the basic principle of multiple testing, stating that the use of such methods undermines the basic premises of empirical research by, among other things, inappropriately penalizing a false significant result above potentially missing a true significant result.

#### Other statistical modeling issues

A collection of other complex statistical issues that arise in analyzing farmworker pesticides is not covered here but should be considered before the analysis. For instance, appropriate statistics for dose–response modeling, lag-time models of washout periods (relevant to recovery), and specific statistics for biomarker analyses all necessitate incorporation of appropriate statistical methods. Even the design and assessment of related questionnaires represent an entire specialty area of statistics and should be given thorough consideration in both the design and analysis phase of the study (Kleinbaum et al. 1998). A detailed discussion of these issues, however, is beyond the scope of this article. Investigators should always involve a statistician in the analysis of such data because even seemingly straightforward analyses may be complicated by any of the above-described factors.

## Statistical Issues Regarding Post Hoc Analyses and Combining Study Results

One issue for potential consideration, in terms of post-hoc analyses, is the area of meta-analysis. Meta-analysis can be defined in terms of either calculating summary measures of effect by synthesizing multiple studies or summarizing variability-of-effect measures across multiple studies (Colditz et al. 1995). In either case, a variety of methods have been outlined to combine results across studies by appropriately modeling the heterogeneity (Greenland 1987).

A related but inherently different issue in post-hoc analyses is the potential for combining study results within a given laboratory or institution, or even across several different laboratories/institutions. The problem may arise for several different reasons. A less-than-significant but still promising result may prompt study investigators to collect further data on additional subjects to boost the statistical power of the resulting study, which leads to several potential complications. One should determine whether additional data can be simply added to the existing data and treated as a single study. This issue can be formally evaluated by examining the variability across and within each individual data set (Evans and Sedransk 2001).

Another issue potentially related to combining a select number of data sets from a given laboratory or set of laboratories is the effect on statistical significance levels. The problem basically leads to repeated testing because we are typically recalculating significance levels at each step without adjusting for these multiple “looks” at the data. This scenario is analogous, or at least related, to sequential testing in clinical trials (Fleming et al. 1984; Green et al. 1987), where we examine significance levels of a given test after enrolling additional patients to the trial. The resulting effect may be relatively minor if the number of looks at the data is small, but the issue should be considered in interpretation of the analyses.

In terms of interpreting statistical tests, related scientific knowledge and the appropriate context for the analysis need to be considered. As an illustration, the direction of the statistical tests should account for *a priori* scientific knowledge. There may be various scenarios where the resulting association can only be in one direction. Associated statistical tests can therefore be more efficient by considering this knowledge and conducting only the appropriate one-sided tests.

## Conclusions

Farmworkers often perform multiple work tasks in different types of agricultural operations. A portion of the farmworker population is highly mobile during the growing season, making it necessary to adopt different approaches and strategies to measure farm-worker exposures. In this article, we discussed important elements of study design and analytical and statistical analysis, with a goal of improving the ability to compare and interpret results across studies performed in different locations and in different segments of the farmworker population. Adequately describing and justifying a study design with regard to sample selection and sample size is imperative in understanding both the aim of the study and the overall results. Hypotheses should be clearly defined. Statisticians should be involved from the beginning to ensure that sample and data collection plans are consistent with the data analysis plan.

Similarly, analytical chemists should be consulted early on to determine the most appropriate analytical techniques to use. Analytical measurements must be carefully selected to ensure that they are both robust and sensitive enough to allow accurate measurement of exposures. The methods used for chemical analysis of both environmental and biological samples should be well characterized so that precision and accuracy in the appropriate concentration ranges are known. Method performance must be carefully monitored with appropriate QC and QA measurements and procedures to generate high-quality and defensible measurement data. Measurement procedures should be selected to minimize missing data and results below LODs, and the approach for dealing with these issues in data analysis should be clearly described. Statisticians should be involved in both the study design and data analysis stages to ensure that issues such as measurement error, multiple testing, and repeat measures are adequately addressed and that appropriate statistical tests and models are used.

Data of high dimensionality will require careful treatment to avoid misrepresenting the significance of the results. In general, researchers should work toward more consistent data reporting with regard to demographic characterization, measurement concentration units, and descriptive statistics. By making transparent both design and analysis procedures, findings among studies can be more reliably compared.
